# Outcome After Surgery for Type A Intramural Hematoma

**DOI:** 10.3390/jcdd13060247

**Published:** 2026-06-03

**Authors:** Fausto Biancari, Angelo M. Dell’Aquila, Timo Mäkikallio, Giuseppe Gatti, Francesco Onorati, Andrea Perrotti, Stefano Rosato, Paola D’Errigo, Matteo Pettinari, Sven Peterss, Joscha Buech, Tatu Juvonen, Caius Mustonen, Till J. Demal, Marek Pol, Petr Kacer, Konrad Wisniewski, Igor Vendramin, Daniela Piani, Mauro Rinaldi, Luisa Ferrante, Eduard Quintana, Robert Pruna-Guillen, Antonio Fiore, Giovanni Mariscalco, Metesh Acharya, Mark Field, Manoj Kuduvalli, Francesco Nappi, Sebastien Gerelli, Dario Di Perna, Javier Rodriguez-Lega, Lenard Conradi

**Affiliations:** 1 Department of Medicine, South-Karelia Central Hospital, University of Helsinki, 53130 Lappeenranta, Finland; 2 Department of Cardiac Surgery, Martin Luther University Halle-Wittenberg, 06108 Halle, Germany; 3 Division of Cardiac Surgery, Cardio-Thoracic and Vascular Department, Azienda Sanitaria Universitaria Giuliano Isontina, 34149 Trieste, Italy; 4 Division of Cardiac Surgery, Trento University Hospital, 38122 Trento, Italy; 5 Department of Cardiovascular and Thoracic Surgery, Dijon University Hospital, 21000 Dijon, France; 6 Center for Global Health, National Health Institute, 00161 Rome, Italy; 7 Cardiac Surgery Unit, Cardiovascular Department, Cliniques Universitaire Saint Luc, 1200 Brussel, Belgium; 8 LMU University Hospital, Ludwig Maximilian University, 81337 Munich, Germany; 9 German Centre for Cardiovascular Research, Partner Site Munich Heart Alliance, 80802 Munich, Germany; 10 Division of Cardiac Surgery, Heart and Lung Center, Helsinki University Hospital, University of Helsinki, 00290 Helsinki, Finland; 11 Research Unit of Surgery, Anesthesia and Critical Care, University of Oulu, 90570 Oulu, Finland; 12 Department of Cardiovascular Surgery, University Heart and Vascular Center Hamburg, 20251 Hamburg, Germany; 13 Department of Cardiac Surgery, Third Faculty of Medicine, Charles University and University Hospital Kralovske Vinohrady, 10000 Prague, Czech Republic; 14 Department of Cardiothoracic Surgery, University Hospital Muenster, 48139 Muenster, Germany; 15 Cardiothoracic Department, University Hospital, 33100 Udine, Italy; 16 Department of Cardiac Surgery, Molinette Hospital, University of Turin, 10126 Turin, Italy; 17 Department of Cardiovascular Surgery, Hospital Clínic de Barcelona, University of Barcelona, 08036 Barcelona, Spain; 18 Department of Cardiac Surgery, Hôpitaux Universitaires Henri Mondor, Assistance Publique-Hôpitaux de Paris, 94000 Creteil, France; 19 Department of Cardiac Surgery, Glenfield Hospital, Leicester LE3 9QP, UK; 20 Liverpool Centre for Cardiovascular Sciences, Liverpool Heart and Chest Hospital, Liverpool L14 3PE, UK; 21 Department of Cardiac Surgery, Centre Cardiologique du Nord de Saint-Denis, 93200 Paris, France; 22 Department of Cardiac Surgery, Centre Hospitalier Annecy Genevois, 74370 Épagny Metz-Tessy, France; 23 Cardiovascular Surgery Department, University Hospital Gregorio Marañón, 28007 Madrid, Spain; 24 Department of Cardiac Surgery, Cologne University Hospital, 50937 Cologne, Germany

**Keywords:** type aortic dissection, aortic dissection, intramural hematoma

## Abstract

**Background:** The nature and prognosis of type A intramural hematoma (TAIMH) are not well established. In this study, we evaluated the early and late outcome after surgery for this emergency condition. **Methods:** The ERTAAD registry included consecutive patients who underwent surgery for acute type A aortic dissection (TAAD) or TAIMH at 18 European centers for cardiac surgery. In this study, we compared the early and late outcomes of TAIMH and typical TAAD. **Results:** In total, 3902 consecutive patients were included in the ERTAAD registry, and 386 (9.9%) patients had TAIMH. Penn class a was present in 43.1% TAAD patients and in 34.5% TAIMH patients (*p* < 0.001). Among 3-month survivors, 10-year relative survival was 0.80 (95%CI 0.77–0.84) in TAAD patients and 0.76 (95%CI 0.63–0.88) in TAIMH patients. Propensity-score matching yielded 386 pairs of patients, and no significant difference was observed between the study groups in terms of early and late outcomes. In-hospital mortality rates were comparable (TAIMH 13.5% vs. TAAD 16.3%, *p* = 0.266). Ten-year mortality was 51.3% among TAIMH patients and 51.6% among TAAD patients (*p* = 0.274). TAIMH and TAAD had similar 10-year cumulative incidence rates of proximal (4.2% vs. 3.3%, *p* = 0.738) and distal aortic reoperations (12.6% vs. 7.6%, *p* = 0.779). **Conclusions:** This cohort study showed that the prevalence of patients with TAIMH requiring surgery is low and their risk profile is significantly different as shown by the Penn classification. The early and late outcomes of the study groups were not statistically different compared to typical TAAD when adjusted for baseline and operative variables. The relative survivals of 90-day TAIMH and TAAD survivors were low compared to the matched general population, indicating that surgically treated TAIMH demonstrated postoperative outcomes are poor and comparable to surgically treated TAAD after adjustment. Post-hoc power analysis suggested that much larger studies are needed to confirm these findings. These results are limited to TAIMH patients who underwent surgery, and this study did not address the outcome of patients who were conservatively treated.

## 1. Introduction

Type A intramural hematoma (TAIMH) is an acute aortic syndrome with the presence of blood of variable amounts with longitudinal extent within the medial layer of the ascending aortic wall, in the absence of an overt intimal tear or patent false lumen on contrast imaging [1]. In a few cases, TAIMH may resorb, but the risk of aneurysm degeneration, aortic dissection, or aortic rupture is significant [1,2]. Indeed, a small series from the International Registry of Aortic Dissection showed that in-hospital mortality for TAIMH was 26.6%, which was comparable to that of type A aortic dissection (TAAD) [3]. However, in-hospital mortality raised to 40% in patients treated conservatively [3]. This has led the Committee of the 2024 ESC Guidelines for the management of peripheral arterial and aortic diseases [1] to suggest urgent surgical repair for TAIMH (class of recommendation I, level of evidence C), while in selected patients with increased operative risk and uncomplicated TAIMH without high-risk imaging features, a “wait-and-see strategy” in an experienced aortic center may be reasonable (class of recommendation IIb, level of evidence C) [1]. On the contrary, reports from Japan and South Korea showed that TAIMH is a more benign condition with favorable early and late outcomes even when treated medically [4,5,6]. It remains unclear whether TAIMH should be considered a condition comparable to TAAD in terms of prognosis. The present European retrospective, multicenter study aimed to evaluate the early and late outcomes of patients who underwent surgery for TAIMH and TAAD.

## 2. Methods

### 2.1. Study Design

This observational, multicenter, retrospective cohort study was performed after approval by the Ethical Review Board of the Helsinki University Hospital, Finland (21 April 2021, diary no. HUS/237/2021) and by the Ethical Review Board of each participating hospital. The requirement for informed consent was waived because of the retrospective nature of this study. The ERTAAD registry included consecutive patients who underwent surgery for acute TAAD or TAIMH at 18 centers for cardiac surgery located in eight European countries (Belgium, Czech Republic, Finland, France, Germany, Italy, Spain, and the United Kingdom) from 1 January 2005, to 31 March 2021 (Table 1). Data were retrospectively collected into a Microsoft Access datasheet (Redmond, WA, USA) with pre-specified baseline, operative, and outcome variables. Data on the date of death and repeated aortic intervention were collected retrospectively from electronic national registries, as well as by contacting regional hospitals, patients, and their relatives.

### 2.2. Study Participants

The study participants were recruited according to the following inclusion criteria: (1) TAAD or TAIMH involving the ascending aorta; (2) patients aged > 18 years; (3) symptoms started within 7 days prior to surgery; (4) primary surgical repair of acute TAAD; and (5) any other major cardiac surgical procedures concomitant with surgery for TAAD [7]. The exclusion criteria were the following: (1) patients aged < 18 years; (2) onset of symptoms > 7 days prior to surgery; (3) previous procedures for TAAD; (4) retrograde TAAD (with primary tear located in the descending thoracic or abdominal aorta); (5) concomitant endocarditis; and (6) TAAD secondary to blunt or penetrating chest trauma [7].

TAIMH was defined as a contained aortic-wall hematoma within the media, without intimal flap formation of the aorta. Patients were considered as having aortic intramural hematoma when no concomitant aortic intimal flap was observed at preoperative aortic imaging. When intramural hematoma was associated with an intimal flap of any other segment of the aorta, patients were classified as having a true aortic dissection.

### 2.3. Urgency of the Procedure

Urgency of the procedure was classified in five grades: (1) urgent, a scheduled procedure performed in paucisymptomatic patients with stable hemodynamic conditions during the index hospitalization since the next working day from admission; (2) emergency grade 1, a procedure performed in patients with stable conditions and without malperfusion before the beginning of the next working day; (3) emergency grade 2, procedure performed in patients with hemodynamic instability despite the use of inotropes and/or any malperfusion before the beginning of the next working day—no cardiopulmonary resuscitation with chest compression required; (4) salvage grade 1, a procedure performed in patients requiring cardiopulmonary resuscitation with external chest compressions and/or open-chest cardiac massage after the induction of anesthesia and before initiation of cardiopulmonary bypass; and (5) salvage grade 2, procedure performed in patients requiring cardiopulmonary resuscitation with external chest compressions *en route* to the operating theater before induction of anesthesia.

### 2.4. Outcome Measures

The primary outcomes of this study were mortality during the index hospitalization, 10-year mortality, and aortic reoperations. Aortic reoperations were classified as proximal procedures when involving the ascending aorta, aortic root, and/or aortic valve. Distal aortic procedures referred to those procedures involving the aorta and/or its branches beyond the ascending aorta. Secondary outcomes were stroke and global brain ischemia, a composite end-point including death, stroke and/or global brain ischemia, paraplegia/paraparesis, tetraplegia/tetraparesis, sepsis, dialysis, heart failure, need of mechanical circulatory support, laryngeal nerve palsy, reoperation for intrathoracic bleeding, tracheostomy, deep sternal wound infection, mesenteric ischemia, limb ischemia, major lower limb amputation, and procedures for intestinal complications. Definition criteria for these outcomes have been previously summarized in the study protocol article of the ERTAAD [7].

### 2.5. Statistical Analysis

**Figure 1:**
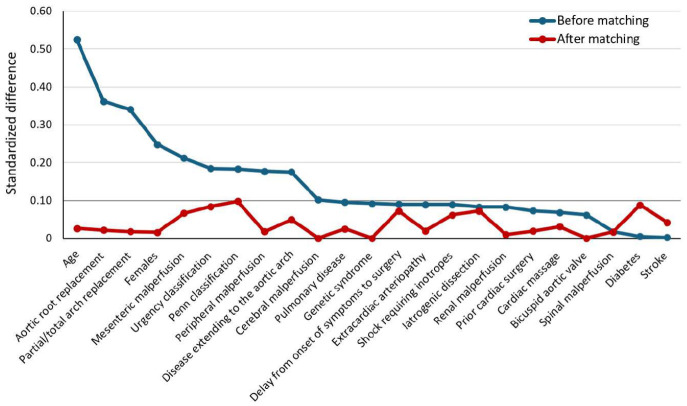
Standardized differences between the cohorts before and after propensity-score matching. The dark gray horizontal line refers to a standardized difference < 0.1, which was considered a non-significant imbalance between the covariates.

Continuous variables were reported as means and standard deviations, and categorical variables as numbers and percentages. Missing values were not replaced. The chi-square test and Fisher’s exact test were used to analyze differences between categorical variables, and the Mann–Whitney test was used to compare continuous variables. Kaplan–Meier’s test with the log-rank test and competing risk analysis with the Fine–Gray test with all-cause death as a competing event were performed for analysis of late mortality and cumulative incidence of aortic reoperations, respectively. Baseline characteristics differed significantly among patients with TAAD and TAIMH. Therefore, propensity score-matching analysis was used to adjust for imbalances between the study groups. A propensity score was estimated using logistic regression, including TAAD and TAIMH, as the dependent variable, considering the following covariates: age; gender; genetic syndrome; bicuspid aortic valve; iatrogenic TAAD; diabetes, stroke; pulmonary disease; extracardiac arteriopathy; prior cardiac surgery; preoperative cardiac massage; cardiogenic shock requiring inotropes; cerebral malperfusion; spinal malperfusion; renal malperfusion; mesenteric malperfusion; peripheral malperfusion; disease extending to the aortic arch/descending thoracic aorta; Penn classification; urgency of the procedure; aortic root replacement; and partial or total aortic arch replacement. Propensity-score matching was performed using the psmatch2 module for Stata, using the nearest-neighbor method with a caliper width of 0.2 the standard deviation of the logit. Standardized difference < 0.1 was considered a non-significant imbalance between the covariates (Figure 1). Country-, age-, and sex-adjusted relative survival was performed using data for the general population retrieved from the Human Mortality Database [8]. Life expectancy of the general population was estimated for each year of the study period according to probabilities of death estimated in the country of each participating hospital. Country-, age-, and sex-adjusted relative survival was estimated using Hakulinen and Tenkkanen’s method. A *p* < 0.05 was set for statistical significance. Statistical analyses were performed with the SPSS (version 27.0, SPSS Inc., IBM, Chicago, IL, USA), SAS (version 9.4, SAS Institute, Cary, NC, USA), and Stata (version 15.1, StataCorp LLC, College Station, TX, USA) statistical software.

## 3. Results

### 3.1. Overall Series

Among 3902 consecutive patients included in the ERTAAD registry, 386 (9.9%) patients had TAIMH. Among the variables imputed into logistic regression, missing values were present in 21 patients (0.005%). The mean follow-up was 3.6 ± 3.6 years (median 2.5) (TAIMH cohort mean of 3.3 ± 3.7 and median of 2.3; TAAD cohort mean of 4.0 ± 4.2 and median of 2.6), and we estimated that 13.2% did not have complete follow-up data; this was mainly due to difficulties in three institutions to gather such data. At 10 years, the rate of censored patients was 54.1% in the TAAD cohort and 49.0% in the TAIMH cohort. The prevalence of TAIMH in the participating hospitals is summarized in Table 1.

Patients with TAIMH were older (mean age, 68.1 vs. 62.8 years, *p* < 0.0001) and were more frequently women (40.9% vs. 29.2%, *p* < 0.0001). There was a balance in most baseline risk factors between TAAD and TAIMH patients (Table 2).

However, TAIMH had lower prevalence of De Bakey type I dissection (78.2% vs. 85.0%, *p* = 0.001), mesenteric malperfusion (1.0% vs. 4.5%, *p* < 0.0001), and peripheral malperfusion (8.8% vs. 14.5%, *p* = 0.002). Penn class a was present in 43.1% TAAD patients and in 34.5% TAIMH patients (*p* < 0.001). The nature of TAIMH led to less frequent aortic root and aortic replacement in TAIMH patients, translating into significantly shorter aortic cross-clamping time (*p* < 0.0001) and cardiopulmonary bypass time (*p* < 0.0001). Standardized differences before and after matching are summarized in Figure 1.

In the overall series, unadjusted mortality was lower in TAIMH patients compared to TAAD patients (*p* = 0.023). TAIMH patients also had a lower risk of stroke/global brain ischemia, mesenteric ischemia, lower limb ischemia, sepsis, dialysis, and reoperation for bleeding. The differences in terms of 10-year outcomes were not statistically significant between the study groups. TAIMH patients were not spared from repeat aortic procedures (Table 3). These procedures were performed in 24 TAIMH patients and the types of aortic reinterventions are listed in Table 4.

### 3.2. Relative Survival Among 90-Day Survivors

**Figure 2:**
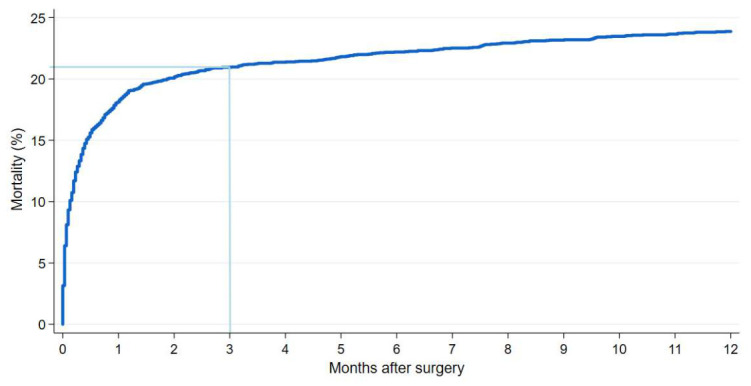
Landmark analysis of early postoperative mortality after surgery for acute type A aortic dissection.

**Figure 3:**
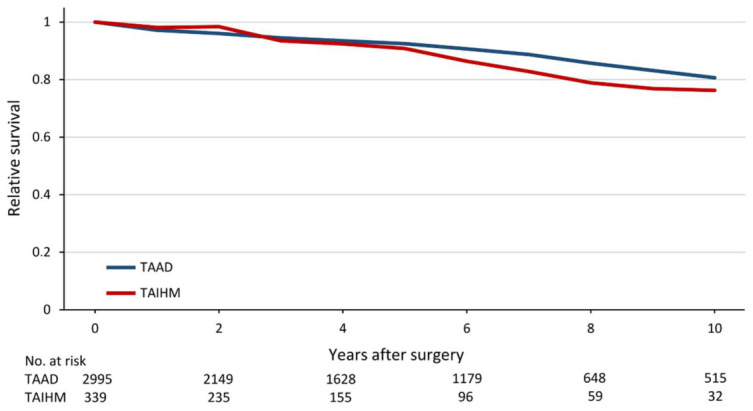
Relative survival in the study groups. TAAD, type A aortic dissection; TAIMH, type A intramural hematoma.

Since early postoperative mortality rate declined about 3 months after surgery, relative survival of the study cohort was estimated among 3-month survivors to exclude any negative prognostic effect of preoperative conditions and surgery on early survival (Figure 2). At 90 days, 2995 TAAD and 339 TAIMH patients were alive. Their 10-year observed survival (66.5% and 59.0%) was markedly lower than their expected survival (82.5% and 77.3%). Such differences yielded relative survivals of 0.80 (95%CI 0.77–0.84) for TAAD patients and 0.76 (95%CI 0.63–0.88) for TAIMH patients (Figure 3).

### 3.3. Propensity Score-Matching Analysis

**Figure 4:**
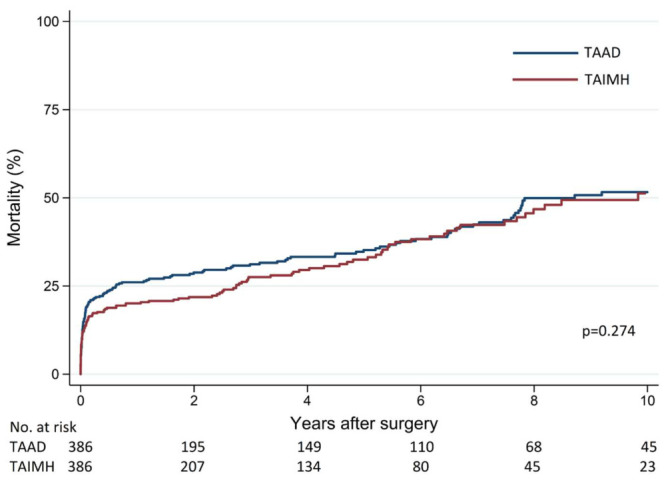
Mortality in propensity score-matched groups. TAAD, type A aortic dissection; TAIMH, type A intramural hematoma.

Propensity-score matching with a caliper weight of 0.16 yielded 386 pairs of patients with balanced preoperative and operative variables, as shown by a standardized difference < 0.10 (Table 2). No significant difference was observed between the study groups in terms of early and late outcomes (Table 3). In-hospital mortality rate in TAIMH patients was not statistically different compared to that of TAAD patients (13.5% vs. 16.3%, *p* = 0.266). Ten-year mortality was 51.3% among TAIMH patients and 51.6% among TAAD patients (*p* = 0.274) (Figure 4). TAIMH and TAAD had similar 10-year incidence rate of proximal (4.2% vs. 3.3%, *p* = 0.738) and distal aortic reoperations (12.6% vs. 7.6%, *p* = 0.779).

## 4. Discussion

The present analysis demonstrated the following: (1) in European countries, the prevalence of TAIMH among surgically treated patients with TAAD was about 10%; (2) TAIMH more frequently affected older patients and women compared to TAAD; (3) TAIMH had lower prevalence of De Bakey type I dissection, mesenteric malperfusion and peripheral malperfusion, and Penn classes b and/c, and it less frequently required aortic root and aortic replacement; (4) when adjusted for baseline and operative confounders, TAIMH patients had similar early and late postoperative outcomes compared to TAAD patients; and (5) among 90-day survivors, 10-year observed survival of both cohorts was lower than the expected age-, sex-, and country-adjusted general population.

Krukenberg was the first to describe in 1920 a case of intramural hematoma [9], and a century later, the nature, prognosis, and optimal management of this acute aortic syndrome are still not well established. Clinicians treating TAIMH face the dilemma of whether to medically or surgically treat a condition which may be potentially benign, with spontaneous resorption of hematoma in a few patients, or be associated with early and late degeneration, leading to typical TAAD and rupture. This controversial issue is partially due to its apparently different behavior in Western and Eastern countries, where epidemiology and prognosis of TAIMH seem to significantly differ. In fact, aortic intramural hematoma is more frequent in Eastern countries, where the disease is more prevalent among women and younger patients, compared to Western countries [10]. Importantly, in Eastern countries, TAIMH is frequently treated medically, and overall, its mortality (medically treated, 4–8%, surgically treated 9–12%) is much lower than in Western countries (medically treated, 33–78%; surgically treated 21–33%). Therefore, investigators from Eastern countries support a policy of medical treatment for uncomplicated TAIMH based on the findings of relatively small studies [10].

Kitai et al. [11] reported on 66 TAIMH patients. Sixteen patients underwent emergency surgery, while 50 patients had medical treatment. Thirty percent of medically treated patients experienced degeneration of the aorta requiring surgery in 13 patients, while two patients who refused surgery died of aortic rupture. Thirty-day mortality was 6% in the emergency surgery group and 4% in the medical therapy group. Later, 16 patients initially medically treated required surgery for progression to TAAD. There were seven late deaths in the medically treated group and none in the emergency surgery group. Overall, at 12-year follow-up, survival in the emergency surgery group was about 90%, and in the medically treated group, it was about 65% (*p* = 0.63).

Choi et al. [4] evaluated the outcome of 61 TAIMH patients, of whom two-thirds were initially treated medically. In-hospital mortality was 7.1% after surgery and 4.3% after medical treatment (*p* = 0.450). Although no statistically significant difference was observed at 2 years, mortality was still 7.1% after surgery, while it increased to 14.9% in the medical group (*p* = 0.450).

Kitamura et al. [5] showed that medical treatment of 46 TAIMH patients resulted in in-hospital mortality of 2% compared to 9% of TAIMH patients who were surgically treated. However, 17% of patients initially medically treated later underwent surgery. In-hospital mortality among those TAIMH patients who declined surgery was 38%. At 3-year, survival was 92% among medically treated patients, 78% among those surgically treated, and 52% among those who declined surgery (*p* = 0.0002).

Song et al. [6] reported on 101 TAIMH patients. Eighty-five patients had only medical treatment, of whom six have died, 62 were discharged alive, and 17 underwent timed operation. This approach resulted in lower in-hospital mortality in TAIMH compared to TAAD patients (7.9% vs. 17.2%, *p* = 0.03). However, mortality did not differ between TAIMH patients who underwent surgery or medical treatment (12.5% vs. 7.1%, *p* = 0.609). Three-year mortality was similar between IMH patients either treated medically or surgically, and TAAD patients (about 83%).

However, less favorable outcomes have been reported with medical treatment also from Eastern centers. Hata et al. [12] described the outcome of 107 TAIMH patients treated surgically with an operative mortality of 3.8%, while 66 patients were treated initially with medical therapy, and their in-hospital mortality was 25.8%. No differences were observed in terms of 10-year survival. Furthermore, according to a pooled analysis by Wee et al. [13], 26.3% of patients of the initially medically treated group required surgical treatment.

In Japan, Matsushita et al. [14] reported on excellent outcome also in surgically treated TAIMH and TAAD with operative mortality rates of 0.9% and 3.4%, respectively (*p* = 0.179). Five-year survival rates were also excellent (93.6% vs. 85.9%, *p* = 0.006). In China, Shi et al. [15] reported an operative mortality rate of 1.9% after delayed surgery for 106 TAIMH patients, with more than half of patients having undergone aortic arch replacement.

Large series of patients are mainly medically treated in Eastern countries [16], and from the available studies, we have learned that maximal aortic diameter >45–50 mm, ulcer-like projections, thickness of hematoma ≥10 mm, and pericardial effusion are characteristics associated with adverse aortic events and may indicate surgery at primary admission [11,16,17]. Still, the unpredictable course of TAIMH [18] leads to considering TAIMH as an emergency condition, which, in most cases, should be treated surgically [1,19]. Indeed, a study from the United States by Estrera et al. [2] showed that among 28 patients with TAIMH who were initially medically treated and later operated on, 12 patients developed typical TAAD. The operative mortality after immediate surgery was 14%, and it was 7% in the delayed surgery group. This study was of small size and therefore was not powered to evaluate whether delayed surgery might be beneficial. However, the risk of conversion from TAIMH to TAAD increased 8 days after presentation, which further confirms the unpredictable nature of aortic intramural hematoma.

Kim et al. [20] showed that among 184 propensity score-matched pairs, TAIMH had significantly better survival at 10 years than typical TAAD (57% vs. 48%, *p* = 0.018) with comparable freedom rates from aortic reoperations (74% vs. 73%, *p* = 0.28). Thirty-day mortality of medically managed TAIMH was 15% versus 8.0% for surgically treated TAIMH, despite similar baseline characteristics [20]. Three-year survival was about 75% among medically managed TAIMH, but analysis was limited to only 25 patients.

Sandhu et al. [21] showed that TAIMH was not associated with significantly decreased early mortality, while its effect in reducing late mortality was significant compared to TAAD (HR, 0.635; 95%CI, 0.407–0.990). However, this study was not adequately powered to answer this issue. To the best of our knowledge, the present analysis is the largest study on the outcome of TAIMH and TAAD and showed that the early mortality and other immediate postoperative adverse events were similarly distributed between the study groups after adjustment for baseline and operative covariates with propensity score-matching analysis. Importantly, the risk of repeat aortic reintervention was not decreased in TAIMH patients compared to TAAD patients. This finding suggested that the aorta of patients with TAIMH is prone to degeneration just as in TAAD patients. This means that the nature of these conditions may be similar, or at least intramural hematoma may be an acute aortic syndrome simply preceding the development of typical features of aortic dissection. Importantly, both early and late mortality rates of this TAIMH series are much higher than reported in Eastern patients. This finding was further confirmed by rather poor relative survival of 90-day survivors. The observation of comparable relative survival among 90-day survivors of the TAIMH and TAAD study groups brought further evidence of the similarities between these two conditions and the mechanisms underlying the poor survival of these patients.

We sought to compare the postoperative results of TAAD and TAIMH patients to evaluate any difference in their outcomes, rather than identify risk factors associated with poor late outcomes. Indeed, evaluation of relative survival at 10 years among patients who survived 3 months after surgery could be of relevance to estimate the outcome of these patients after recovery from surgery compared to country-, age-, and sex-matched subjects of the general population. In this regard, it is of utmost importance to investigate whether aortic-related and/or systemic conditions are the reasons for such poor late relative survival in both TAAD and TAIMH patients. At this stage, the finding of poor relative survival reinforces the need for long-term surveillance in these patients.

TAIMH may be regarded as a condition with less benign presentation of TAIMH compared to TAAD. Still, aortic intramural hematoma can be associated with signs and symptoms of malperfusion, aortic valve regurgitation, and hemorrhagic pericardial and pleural effusion also in other studies [3,6,20,22,23]. In this study, the decision of surgical repair in these patients was likely substantiated by the fact that the mean diameter of the ascending aorta was similar in TAAD and TAIMH patients, and by the presence of pericardial effusion in almost half of patients with TAIMH, along with other dissection-related conditions (Table 2). In the IRAD registry, 15% of patients with TAIMH presented with pulse deficits, and 12% were hypotensive [3]. Malperfusion is likely secondary to the stenosing effect of intramural hematoma on the aortic branches that is observed in typical TAAD [24]. Importantly, the present study included only patients who underwent surgery. Therefore, we dealt with patients who underwent surgery based on clinical symptoms, which may partly explain the differences in the prevalence of clinical variables with studies including also patients who underwent medical management.

A previous study from this registry showed interinstitutional differences in terms of patients’ characteristics, referral timing, and surgical approaches [25]. This might have a significant impact on the results of this study, but the small number of patients with TAIMH prevents sensitivity analysis or multilevel regression analyses to estimate the effect of interinstitutional differences in terms of postoperative outcomes. However, relative survival estimates considered the effect of each country and likely provided reliable information on the expected/observed survival of these patients.

The retrospective nature of this study is its main limitation. Second, data on late mortality and aortic reoperations were gathered from control visits; from electronic national registries; and by contacting regional hospitals, patients, and their relatives. However, its completeness cannot be verified. Indeed, in some countries, there are difficulties gathering long-term follow-up, while in other countries, data on mortality and reoperations were available for nearly all patients. Furthermore, many patients operated on for acute TAAD are often temporary immigrants and tourists, and their follow-up is not feasible. Sensitivity analysis including patients operated at the Helsinki Central Hospital, Finland, with complete follow-up data demonstrated that the TAAD and TAIMH had similar late survival, as adjusted for multiple variables (HR, 0.810; 95%CI, 0.407–1.613). Third, the prevalence and severity of both TAAD and TAIMH might vary between the participating hospitals, because of different referral pathways and distances from first- or second-level referral centers. Fourth, we do not have data on patients who were treated conservatively. This prevents an analysis of the characteristics of patients medically and those surgically treated, as well as their outcomes. Fifth, we do not have data either on the size of the thoracic aortas or on the thickness of the aortic hematoma to verify that TAIMH patients were operated on because of a high risk of aortic-related events. Sixth, this analysis does not include patients with TAIMH who were treated conservatively. Therefore, the present findings do not allow for a thorough analysis of the early and late prognosis of TAIMH or identify the characteristics which indicate surgical repair. Seventh, early and late outcomes were adjusted also for the urgency of the procedure; the extent of surgery; and other operative characteristics which are treatment-mediated variables, not true baseline confounders. However, these variables reflect the extent of surgery, as well as the need for prompt surgical treatment. Still, these factors have significant prognostic implications, and excluding them could certainly introduce bias in comparative analysis of the early and late outcome. Finally, despite the relatively large number of patients of this multicenter registry, post hoc analysis showed that, to reject the null hypothesis (overall in-hospital mortality rates, 18.1% vs. 13.5%; alpha, 0.05; beta, 0.80), this analysis would have required 986 patients in each study group. Therefore, this study was not powered to reach conclusive results on the possibly reduced risk of early mortality in patients with TAIMH.

## 5. Conclusions

This cohort study showed that the prevalence of patients with TAIMH requiring surgery is low and their risk profile is significantly different, as shown by the Penn classification. Their early and late outcomes were not statistically different compared to typical TAAD when adjusted for baseline and operative variables. The relative survivals of 90-day TAIMH and TAAD survivors were low compared to the matched general population, indicating that surgically treated TAIMH demonstrated postoperative outcomes that are poor and comparable to surgically treated TAAD after adjustment. Post-hoc power analysis suggested that much larger studies are needed to confirm these findings. It is worth noting that these results are limited to TAIMH patients who underwent surgery, and this study did not address the outcome of patients who were conservatively treated.

## Figures and Tables

**Table 1:** Patients and their in-hospital and 10-year all-cause mortality in the participating hospitals.

No.	Participating Hospitals	Overall No. of Patients (%)	No. of Patients with Intramural Hematoma (%)	In-Hospital Mortality (%)	10-Year Mortality (%)
1	A	132 (3.4)	14 (10.6)	18.9	41.4
2	B	156 (4.0)	12 (7.7)	16.7	46.3
3	C	249 (6.4)	24 (9.6)	18.5	46.5
4	D	69 (1.8)	1 (1.4)	27.5	45.9
5	E	281 (7.2)	35 (12.5)	17.1	37.9
6	F	329 (8.4)	34 (10.3)	13.7	38.3
7	G	133 (3.4)	26 (19.5)	19.4	54.6
8	H	172 (4.4)	14 (8.1)	11.6	53.1
9	I	105 (2.7)	17 (16.2)	27.6	47.6
10	L	341 (8.7)	25 (7.3)	12.9	34.5
11	M	308 (7.9)	29 (9.4)	17.9	52.2
12	N	167 (4.3)	16 (9.6)	25.1	44.3
13	O	293 (7.5)	11 (3.8)	27.0	44.3
14	P	81 (2.1)	9 (11.1)	30.9	75.4
15	Q	492 (12.6)	68 (13.8)	18.9	51.8
16	R	141 (3.6)	12 (8.5)	17.7	51.9
17	S	182 (4.7)	18 (9.9)	9.9	54.3
18	T	271 (6.9)	21 (7.7)	8.9	57.2

**Table 2:** Patients’ characteristics and operative data of unmatched and propensity score-matched cohorts.

	Unmatched Cohorts			Propensity Score-Matched Cohorts		
	TAAD No. = 3516	TAIMH No. = 386	Standardized Differences	TAAD No. = 386	TAIMH No. = 386	Standardized Differences
Age, mean (SD), y	62.8 (13.0)	68.1 (12.1)	0.525	67.8 (11.9)	68.1 (12.1)	0.026
Females, no. (%)	1027 (29.2)	158 (40.9)	0.248	155 (40.2)	158 (40.9)	0.016
Genetic syndrome, no. (%)	77 (2.2)	4 (1.0)	0.092	4 (1.0)	4 (1.0)	0.000
Bicuspid aortic valve, no. (%)	140 (4.0)	11 (2.8)	0.062	11 (2.8)	11 (2.8)	0.000
Iatrogenic dissection, no. (%)	97 (2.8)	6 (1.6)	0.083	3 (0.8)	6 (1.6)	0.072
Diabetes, no. (%)	177 (5.0)	19 (4.9)	0.005	27 (7.0)	19 (4.9)	0.088
Stroke, no. (%)	138 (3.9)	15 (3.9)	0.002	12 (3.1)	5 (3.9)	0.042
Pulmonary disease, no. (%)	285 (4.9)	42 (10.9)	0.095	39 (10.1)	42 (10.9)	0.025
Extracardiac arteriopathy, no. (%)	172 (4.9)	27 (7.0)	0.089	29 (7.5)	27 (7.0)	0.020
Prior cardiac surgery, no. (%)	114 (3.2)	8 (2.1)	0.073	7 (1.8)	8 (2.1)	0.019
Cardiac massage, no. (%)	155 (4.4)	12 (3.1)	0.068	10 (2.6)	12 (3.1)	0.031
Shock requiring inotropes, no. (%)	595 (16.9)	53 (13.7)	0.089	45 (11.7)	53 (13.7)	0.062
Cerebral malperfusion, no. (%)	782 (21.7)	68 (17.6)	0.102	68 (17.6)	68 (17.6)	0.000
Spinal malperfusion, no. (%)	73 (2.1)	9 (2.3)	0.017	10 (2.6)	9 (2.3)	0.017
Renal malperfusion, no. (%)	336 (9.6)	28 (7.3)	0.083	27 (7.0)	28 (7.3)	0.010
Mesenteric malperfusion, no. (%)	158 (4.5)	4 (1.0)	0.212	7 (1.8)	4 (1.0)	0.066
Peripheral malperfusion, no. (%)	509 (14.5)	34 (8.8)	0.177	36 (9.3)	34 (8.8)	0.018
Disease extending to the aortic arch/descending thoracic aorta, no. (%)	2973 (85.0)	302 (78.2)	0.175	294 (76.2)	302 (78.2)	0.049
Diameter of the ascending aorta, mean (SD), mm *	51 (10)	50 (8)	0.043	51 (11)	50 (10)	0.065
Aortic valve insufficiency, no. (%) **			0.512			0.378
No/trace	1458 (41.8)	210 (55.0)		187 (48.7)	210 (55.0)	
Mild	751 (21.5)	114 (29.8)		82 (21.4)	114 (29.8)	
Moderate	589 (16.9)	32 (8.4)		53 (13.8)	32 (8.4)	
Severe	689 (19.8)	26 (6.8)		62 (16.1)	26 (6.8)	
Pericardial effusion, no. (%) ***	945 (30.6)	172 (47.4)	0.349	117 (30.3)	181 (46.9)	0.345
Penn classification, no. (%)			0.183			0.098
a	2000 (56.9)	253 (65.5)		256 (66.3)	253 (65.5)	
b	1207 (34.3)	159 (41.2)		81 (21.0)	159 (41.2)	
c	239 (6.8)	24 (6.2)		16 (4.1)	24 (6.2)	
b+c	389 (11.1)	34 (8.8)		33 (8.5)	34 (8.8)	
Urgency classification, no. (%)			0.184			0.084
Urgent	551 (15.7)	68 (17.6)		65 (16.8)	68 (17.6	
Emergency 1	1207 (34.3)	159 (41.2)		150 (38.9)	159 (41.2)	
Emergency 2	1592 (45.3)	146 (37.8)		160 (41.5)	146 (37.8)	
Salvage 1	102 (2.9)	7 (1.8)		7 (1.8)	7 (1.8)	
Salvage 2	64 (1.8)	6 (1.6)		4 (1.0)	6 (1.6)	
Delay from onset of symptoms to surgery, mean (SD), hours	15 (24)	18 (23)	0.090	16 (24)	18 (23)	0.072
Operative data						
Intimal tear site, no. (%)						
Aortic root	635 (18.1)	55 (14.2)	0.104	58 (15.0)	55 (14.5)	0.021
Ascending aorta	2248 (63.9)	246 (63.7)	0.004	286 (74.1)	246 (63.7)	0.225
Aortic arch	587 (16.7)	59 (15.3)	0.004	34 (8.8)	59 (15.3)	0.199
Partial/total arch replacement, no. (%)	741 (21.1)	35 (9.1)	0.340	33 (8.5)	35 (9.1)	0.018
Aortic root replacement no. (%)	1040 (29.6)	57 (14.8)	0.362	54 (14.0)	57 (14.8)	0.022
XCT time, mean (SD), min	122 (60)	101 (48)	0.391	109 (54)	101 (48)	0.165
CPB time, mean (SD), min	219 (90)	187 (78)	0.381	201 (82)	187 (78)	0.168

Abbreviations: CPB = cardiopulmonary bypass; SD = standard deviation; XCT = aortic cross clamping time. * Data available from computed tomography measurements in 1944 patients with TAAD and 233 patients with TAIMH. ** Data available in 3487 patients with TAAD and 382 patients with TAIMH. *** Data available in 3087 patients with TAAD and 363 patients with TAIMH who underwent preoperative computed tomography.

**Table 3:** Outcomes in unmatched and propensity score-matched cohorts.

	Unmatched Cohorts			Propensity Score-Matched Cohorts		
	TAAD No. = 3516	TAIMH No. = 386	*p*-Value	TAAD No. = 386	TAIMH No. = 386	*p*-Value
**Early outcomes**						
Hospital mortality, no. (%)	637 (18.1)	52 (13.5)	0.023	63 (16.3)	52 (13.5)	0.266
Stroke/global brain ischemia, no. (%)	670 (19.1)	53 (13.7)	0.011	63 (16.3)	53 (13.7)	0.314
Composite endpoint, no. (%)	1066 (30.3)	86 (22.3)	0.001	99 (25.6)	86 (22.3)	0.273
Paraparesis/paraplegia	189 (5.4)	15 (3.9)	0.277	22 (5.7)	15 (3.9)	0.238
Tetraparaparesis/tetraparaplegia, no. (%)	3 (0.1)	0 (0)	1.000	0 (0)	0 (0)	-
Mesenteric ischemia, no. (%)	144 (4.1)	5 (1.3)	0.005	13 (3.4)	5 (1.3)	0.092
Sepsis, no. (%)	440 (12.5)	34 (8.8)	0.033	46 (11.9)	34 (8.8)	0.156
Dialysis, no. (%)	526 (15.0)	33 (8.5)	<0.0001	41 (10.6)	33 (8.5)	0.328
Laryngeal nerve injury, no. (%)	65 (1.8)	6 (1.6)	0.681	3 (0.8)	6 (1.6)	0.505
Tracheostomy, no. (%)	294 (8.4)	25 (6.5)	0.240	26 (6.7)	25 (6.5)	0.885
Reoperation for bleeding, no. (%)	513 (14.6)	36 (9.3)	0.004	50 (13.0)	36 (9.3)	0.137
Deep sternal wound infection, no. (%)	81 (2.3)	8 (2.1)	0.773	5 (1.3)	8 (2.1)	0.401
Heart failure, no. (%)	508 (14.4)	44 (11.4)	0.103	49 (12.7)	44 (11.4)	0.580
MCS, no. (%)	128 (3.6)	13 (3.4)	0.785	9 (2.3)	13 (3.4)	0.387
Acute lower limb ischemia, no. (%)	119 (3.4)	5 (1.3)	0.022	7 (1.8)	5 (1.3)	0.773
Major lower limb amputation no. (%)	17 (0.5)	0 (0)	0.402	1 (0.3)	0 (0)	1.000
Acute upper limb ischemia, no. (%)	11 (0.3)	2 (0.5)	0.373	0 (0)	2 (0.5)	0.499
Surgery for intestinal complications, no. (%)	18 (0.5)	0 (0)	0.247	2 (0.5)	0 (0)	0.499
**10-year outcomes**						
Mortality, (%)	47.6	51.3	0.491	51.6	51.3	0.274
Distal aortic reoperation, (%)	7.2	12.6	0.886	7.6	12.6	0.779
Proximal aortic reoperation, (%)	4.3	4.2	0.425	3.3	4.2	0.738

Composite endpoint = in-hospital death, stroke, and/or global brain ischemia. Abbreviation: MCS = mechanical circulatory support, i.e., intra-aortic balloon pump and/or extracorporeal membrane oxygenation.

**Table 4:** Late repeat aortic procedures in 24 patients with TAIMH.

Aortic Procedure	No.
TEVAR	11
EVAR	3
Frozen elephant trunk	3
Supracoronary aortic repair	3
Open repair of the abdominal aorta	2
AVR	2
Bentall procedure	2
Bentall procedure, frozen elephant trunk	1
Bentall procedure, total aortic arch repair	1
Open repair of the descending thoracic aorta	1
Total	30

Abbreviations: AVR = aortic valve replacement; EVAR = endovascular abdominal aortic repair; TEVAR = thoracic endovascular aortic repair.

## Data Availability

The dataset presented in this article is not available because of privacy issues.
